# A Novel Algorithm for Adaptive Detection and Tracking of Extended Targets Using Millimeter-Wave Imaging Radar

**DOI:** 10.3390/s25103029

**Published:** 2025-05-11

**Authors:** Ge Zhang, Weimin Shi, Xiaofeng Shen, Qilong Miao, Chenfei Xie, Lu Chen

**Affiliations:** 1 School of Information and Communication Engineering, University of Electronic Science and Technology of China, Chengdu 611731, China; zhangge@std.uestc.edu.cn (G.Z.); 201911012021@std.uestc.edu.cn (Q.M.); 2 School of Microelectronics and Communication Engineering, Chongqing University, Chongqing 400044, China; wmshi@cqu.edu.cn; 3 National Key Laboratory of Wireless Communications, University of Electronic Science and Technology of China, Chengdu 611731, China; chenff93625@163.com; 4 School of Aeronautics and Astronautics, University of Electronic Science and Technology of China, Chengdu 611731, China; lchen@std.uestc.edu.cn

**Keywords:** high-resolution imaging radar, extended target, scattering point shift, adaptive detection and tracking

## Abstract

A high-resolution imaging radar is exceptionally well-suited for the detection and perception of extended targets (ETs), as it provides a comprehensive representation of the spatial distribution of target scattering characteristics. In this work, we propose an adaptive detection and tracking framework for non-cooperative ETs based on radar imaging. The framework leverages the statistical properties of ETs in radar imaging to construct a target distribution model and introduces an adaptive ET detection and tracking algorithm based on Scattering Point Shift (SPS). This algorithm is designed to track ETs with internal motion characterized by multiple scattering points. The initial target distribution is estimated using two-dimensional kernel density estimation (2D-KDE). Compared to existing ET tracking algorithms, the proposed SPS method demonstrates superior universality in accommodating diverse scattering point distributions and integrates detection and tracking into a unified process, thereby significantly improving information utilization efficiency. The effectiveness of the algorithm is validated through extensive simulations and real-world data collected using a millimeter-wave (mmWave) imaging radar operating in the Linear Frequency Modulated Continuous Wave (LFMCW) mode.

## 1. Introduction

Recent advancements in hardware technology and the exponential growth of computational power have facilitated the widespread adoption of high-bandwidth mmWave imaging radar across diverse applications, including autonomous driving, road surveillance, and security monitoring [1,2,3]. In these operational contexts, the high bandwidth and large aperture of radar systems enable high-resolution imaging, resulting in most targets being characterized as ETs comprising multiple scattering points [4,5,6]. Within the realm of radar signal processing, ET tracking and detection are of paramount importance, significantly contributing to improved situational awareness and decision-making precision [7,8]. As a result, there is an imperative need to develop accurate and robust tracking and detection methodologies tailored for ETs.

In contrast to point targets, ETs occupy multiple resolution cells in radar measurements, resulting in highly complex scattering characteristics [9,10]. For instance, ETs such as large vehicles, groups of pedestrians, or multi-target formations exhibit intricate shapes, varying sizes, and dynamic motion patterns, which often exceed the capabilities of non-adaptive radar processing algorithms [11]. This inherent complexity demands advanced algorithms that can precisely estimate target extent, orientation, and dynamic behavior. Traditional tracking methods, including the Kalman Filter (KF) and its derivatives, are frequently inadequate for ETs, as they rely on the assumption of point-like scattering centers. To address these challenges, researchers have developed specialized techniques, such as Random Finite Set (RFS) theory, Bayesian frameworks, and machine learning-based approaches [12]. Nevertheless, despite notable advancements, existing methods still face several limitations that impede their effectiveness in practical applications.

Early tracking methodologies predominantly relied on model-based techniques, which assumed that the shape and size of ETs were known a priori [13]. These approaches utilized point target tracking algorithms, such as the KF [14] and particle filter (PF) [15], to achieve target tracking. However, such methods frequently proved inadequate in addressing the complexities associated with unknown, non-cooperative ETs, leading to significant inaccuracies in both detection and tracking performance.

Recent advancements in signal processing have predominantly focused on the development of algorithms specifically designed to address the extended nature of targets. Among the most significant breakthroughs in this domain is the introduction of the Extended Target Tracking (ETT) framework. This innovative model extends beyond the conventional point-target paradigm by representing targets as either a collection of discrete points or extended shapes (e.g., Gaussian or elliptical distributions) within the radar observation space, thereby effectively accommodating the spatial distribution characteristics of targets [16,17].

RFS-based approaches, particularly the Probability Hypothesis Density (PHD) filter and the Cardinalized PHD (CPHD) filter, have been extensively employed in ET tracking applications [18,19]. These methodologies model both target states and measurements as random finite sets, thereby providing a robust framework for handling varying numbers of targets and measurements. For instance, ref. [20] developed a Gaussian Mixture PHD filter specifically tailored for ETs, demonstrating superior performance in cluttered environments. Similarly, ref. [21] introduced the Labeled Multi-Bernoulli filter, which enhances tracking accuracy through the integration of target identity information. Despite these advancements, these methods often encounter challenges related to computational complexity, particularly in dynamic target scenarios, and may exhibit limitations in effectively capturing temporal variations in target morphology.

Bayesian methodologies employ probabilistic models to estimate target states and extents [22,23]. In this context, ref. [24] introduced a hierarchical Gaussian process-based Bayesian algorithm for ET tracking, which effectively captures temporal variations in target morphology. Furthermore, ref. [25] developed a variational Bayesian approach for joint tracking and classification of ETs, demonstrating robust performance in multi-target scenarios. While these methods exhibit theoretical rigor, they are inherently computationally intensive and often require substantial prior knowledge, which may limit their applicability in non-cooperative target environments.

Recent advancements in machine learning have introduced novel solutions for ET tracking [26]. Notably, ref. [27] developed a Convolutional Neural Networks (CNN)-based approach for target extent estimation, achieving high precision in complex environments. Similarly, ref. [28] proposed a Recurrent Neural Network (RNN) framework for dynamic target tracking, demonstrating significant improvements in tracking continuity and accuracy. Despite these promising developments, these methods typically require extensive labeled training datasets, which may not always be readily available. Furthermore, their performance tends to degrade in scenarios with substantial environmental variations, and their computational intensity often poses challenges for real-time applications.

Despite these advancements, significant challenges persist in the detection and tracking of ETs. A primary concern lies in the accurate modeling of both the shape and motion characteristics of ETs, particularly in complex environments where targets exhibit highly dynamic behavior [29,30].

In this work, we present a novel adaptive detection and tracking framework for non-cooperative ETs based on mmWave imaging radar. Our contributions are summarized as follows:

- Utilizing the statistical properties of ETs in radar imaging, a two-dimensional Gaussian mixture model (2D-GMM) is constructed to represent the target distribution. The target distribution information is extracted from the radar images generated in each coherent processing interval (CPI) through a 2D-KDE approach. This model is highly applicable to most targets in high-resolution radar sensing scenarios and provides an accurate representation of the target scattering distribution characteristics.
- We propose an adaptive ET detection and tracking algorithm based on scattering point shift (SPS). In addition to rigid targets, this algorithm can effectively handle complex targets with independently moving internal modules, demonstrating a robust performance. By simultaneously performing tracking and detection, the proposed algorithm achieves higher efficiency and lower information loss compared to traditional track-after-detect approaches.
- The effectiveness of the proposed algorithm is jointly validated through simulations and field measurements, demonstrating its feasibility for practical applications.

The rest of this article is organized as follows. Section 2.1 elaborates on the signal model of the LFMCW mmWave imaging radar and the back-projection algorithm in radar imaging, accompanied by mathematical formulations to illustrate the input data for the proposed algorithm. Section 2.2 constitutes the core of the proposed methodology, where we first discuss the target distribution model within the framework of our algorithm, followed by the derivation of three key components: the target model initialization algorithm, the ET detection and tracking algorithm based on SPS, and the likelihood ratio detection algorithm. Section 3 evaluates the algorithm’s performance metrics through comprehensive simulation experiments and validates the framework’s practicality using real-world measurements. Finally, Section 4 concludes the paper with a summary of the overall work and its contributions.

## 2. Materials and Methods

### 2.1. Signal Model and Algorithm Framework

#### 2.1.1. LFMCW Radar Echo Signal Model

A 77 GHz millimeter-wave imaging radar (University of Electronic Science and Technology of China, Chengdu, China) employing a linear frequency-modulated continuous wave as the transmitted signal was utilized to acquire and process target echoes. In the context of detecting ETs characterized by multiple scattering points, the baseband signal obtained after receiver down-conversion can be represented as:

(1) $$S_{b}\left(t,b,l\right)=\sum_{m=1}^{M}\exp\{j2\pi \left[f_{0}\tau_{m}+K\tau_{m}t+f_{d,m}bT+\frac{ldsin\theta_{m}}{\lambda }\right]\}$$

In this formulation, *M* denotes the total number of scattering points, and *m* represents their respective index. The fast-time sampling sequence is denoted by *t*, while *b* corresponds to the pulse index in coherent processing. The index of the virtual receiving array element in the 77 GHz MIMO radar is represented by *l*. Additionally, *K* signifies the frequency modulation slope, $f_{0}$ is the carrier frequency, and $\tau_{m}$ denotes the two-way delay of the *m*-th scattering point. The Doppler frequency of the m-th scattering point is given by $f_{d,m}$, *T* is the pulse repetition interval, λ is the wavelength, *d* represents the spacing between antenna array elements, and $\theta_{m}$ indicates the azimuth angle of the *m*-th scattering point.

The radar baseband echo signal, after undergoing matched filter processing, yields a one-dimensional range profile of the imaging area, which reflects the scattering distribution of targets within the beam along the range dimension.

(2) $${\tilde{S}}_{b}\left(t,b,l\right)=\int_{-\infty }^{\infty }S_{b}\left(\tau ,b,l\right)\cdot ref\left(\tau -t\right)d\tau ,$$

The complex conjugate of the transmitted signal, denoted as ref(t), is utilized as the reference signal for matched filtering. This process provides significant processing gain to the signal, thereby enhancing the signal-to-noise ratio (SNR).

#### 2.1.2. Backprojection Imaging

The radar imaging process is typically accomplished through joint processing using either an antenna array or a network of multiple radar sensors. In our approach, we employ a sensor network composed of multiple millimeter-wave radars to simultaneously collect the target’s echo signals. After processing, a one-dimensional range profile matrix H of the imaging region is obtained.

Each element in the one-dimensional range profile matrix H represents the integral of scattering points within the same range bin across the angular dimension of the sensor beam:

(3) $$H\left(t,n\right)=\int_{x}\int_{y}\int_{z}\sigma \left(p\right)e^{-j2\pi f_{0}\tau_{t,n}}d_{x}d_{y}d_{z},\tau_{t,n}=2r_{p,n}/c,$$

its discrete representation can be expressed as:

(4) $$H\left(t,n\right)=\sum_{P}\sigma \left(p\right)e^{-j2\pi f_{0}\tau_{t,n}},\tau_{t,n}=2r_{p,n}/c,$$

σ(p) denotes the scattering intensity at a specific position p within the imaging region.

The back-projection imaging algorithm is widely employed in radar imaging due to its advantages of low computational complexity and strong applicability to diverse detection scenarios. The fundamental principle of the back-projection imaging algorithm lies in mapping echoes from multiple incidence angles onto the imaging space of the detection region through a back-projection process, followed by coherent superposition to generate the radar image. Since the signal channels corresponding to the spatial units containing scattering points achieve phase coherence after time-delay compensation, maximum gain is attained, resulting in high-amplitude pixels in the radar image. By applying back-projection to H, the image of the imaging region *P* is obtained:

(5) $$I\left(p\right)=\sum_{n=1}^{N}H\left(t+\tau_{p,n},n\right)=\sum_{n=1}^{N}H(t,n)\cdot e^{j2\pi f_{0}\tau_{p,n}},p\in P,$$

The amplitude of individual pixels in the image exhibits a direct correspondence with the scattering intensity of the target’s constituent points:

(6) $$I\left(x,y\right)=I\left(p\right)=\hat{\sigma }\left(p\right),p=\left(x,y\right)\in P,$$

p denotes the positional coordinates (x,y) of a pixel, while $\hat{\sigma }\left(p\right)$ represents the observed scattering intensity σ(p) at the location p.

#### 2.1.3. Algorithm Framework

**Figure 1:**
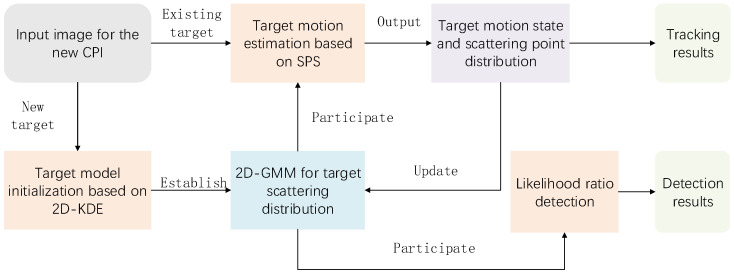
Framework of the adaptive detection and tracking algorithm for ETs.

Figure 1 presents the comprehensive framework of the adaptive detection and tracking algorithm for ETs utilizing a millimeter-wave imaging radar. The algorithm’s input consists of the imaging results derived from the radar’s echo signal processing of the target region. New targets and existing targets are processed independently. For new targets, an initial two-dimensional Gaussian mixture model is established based on 2D-KDE. For existing targets, the distribution and motion state of the scattering points are estimated and updated using the scattering point drift algorithm. Throughout the process, the algorithm concurrently performs detection and tracking of ETs.

### 2.2. Adaptive Detection and Tracking Algorithm for ETs

#### 2.2.1. 2D-GMM for ETs

Following matched filtering, the amplitude values of the scattering points corresponding to the extended target in the one-dimensional range profile are characterized by the point spread function:

(7) $$H\left(t,n\right)=\sum_{m=1}^{M}A_{m}\sin c\left(B\cdot \left(t-\tau_{m,n}\right)\right),$$

$\tau_{m,n}$ denotes the two-way delay of the *m*-th scattering point in the *n*-th angle echo, and *B* represents the signal bandwidth. Consequently, the range resolution Δr of the echo can be expressed as:

(8) Δr=c/2B

Upon performing back-projection imaging, the amplitude values of the extended target in the two-dimensional image conform to a two-dimensional Gaussian distribution. 2D-GMM demonstrates superior applicability for robust target characterization due to its capability of smooth approximation of arbitrary shapes and multimodal histograms [31,32]. The mixture parameters—including mean vectors, covariance matrices, and component weights—collectively form a comprehensive feature vector. Thus, for an extended target comprising multiple scattering points with independent intensity and distribution properties, its two-dimensional image distribution can be effectively modeled as a two-dimensional Gaussian mixture model.

(9) $$Gmm\left(p\right)=\sum_{m}^{M}\sigma_{m}N\left(p|{\bar{p}}_{m},R_{m}\right),$$

$N(p|{\bar{p}}_{m},R_{m})$ represents the two-dimensional distribution of the *m*-th scattering point, ${\bar{p}}_{m}$ corresponds to its center, $R_{m}$ describes its distribution covariance matrix, and $\sigma_{m}$ is related to its weight and scattering point intensity. The expansion of can be expressed as follows:

(10) $$N\left(p,|{\bar{p}}_{m},R_{m}\right)=\frac{\exp\left[-\frac{1}{2}{\left(p-{\bar{p}}_{m}\right)}^{T}{\left(R_{m}\right)}^{-1}\left(p-{\bar{p}}_{m}\right)\right]}{2\pi {\left|R_{m}\right|}^{\frac{1}{2}}}$$

In the two-dimensional image, the integrated normalized amplitude $\bar{I}\left(p\right)$ at each pixel location p serves as an indicator of the “occurrence rate,” reflecting the frequency of its appearance.

(11) $$\bar{I}\left(p\right)=\frac{I(p)}{\sum_{q}^{Q}I(q)}$$

#### 2.2.2. Target Model Initialization Based on 2D-KDE

For newly detected targets, an initial two-dimensional Gaussian mixture distribution model must be constructed. Since the number of target scattering points—or more precisely, the number of scattering points with intensities within the range of interest—is unknown, it is essential to fit a two-dimensional distribution curve to determine the number of mixture components. In the two-dimensional image, the presence of non-smooth connected pixels resulting from discrete processing significantly impacts the estimation of model parameters.

To overcome these challenges, a two-dimensional kernel density estimation approach is employed to fit the distribution curve, enabling the estimation of both the number of mixture components and their corresponding parameters.

By utilizing a two-dimensional Gaussian kernel function, the probability density $\hat{f}\left(p\right)$ at position p is computed as follows:

(12) $$\hat{f}\left(p\right)=\sum_{q}^{Q}\bar{I}\left(q\right)\cdot \frac{1}{2\pi h^{2}}\cdot \exp\left\{-\frac{{∥p-q∥}^{2}}{2h^{2}}\right\}$$

The window length *h* of the 2D Gaussian kernel critically determines the smoothness of the density estimation. For high-resolution radar images, we recommend initializing *h* as twice the range resolution Δr, followed by empirical fine-tuning based on actual data characteristics.

Normalization is performed as follows:

(13) $$f\left(p\right)=\frac{\hat{f}\left(p\right)}{\sum_{q}^{Q}\hat{f}\left(q\right)}$$

Once the two-dimensional distribution curve is obtained, the set of peak points ${\tilde{p}}_{K}$ is extracted, and the number of peak points is determined as the number of mixture components, denoted by *K*.

By employing 2D-KDE, the number of models, along with the initial centers and weights of the mixture models, can be derived. To construct an effective target model, accurate estimation of three key parameters weights $\sigma_{K}$, centers ${\bar{p}}_{K}$, and distribution covariance matrices $R_{K}$ is essential. The workflow of the estimation algorithm is outlined below:

1. Initialize the parameters: ${\sigma_{K}}^{0}$ corresponds to the normalized amplitudes $I({\tilde{p}}_{k})$ of the *K* peak points in the image, ${{\bar{p}}_{K}}^{0}$ represents ${\tilde{p}}_{K}$, the positions of these peak points, and ${R_{K}}^{0}$ is initialized based on the range resolution Δr and angular resolution Δθ;
2. The iterative process begins, with *i* serving as the iteration counter;
3. The probability ${\hat{g}}_{k}\left(p\right)$ that all imaging region units belong to each scattering point model is computed. Here, ${\hat{g}}_{k}\left(p\right)$, acting as a latent variable in the model, quantifies the membership degree of each data point within the Gaussian component:
  (14) $${\hat{g}}_{k}\left(p\right)=\frac{{\sigma_{k}}^{i-1}N\left(p|{{\bar{p}}_{k}}^{i-1},{R_{k}}^{i-1}\right)}{\sum_{k}^{K}{\sigma_{k}}^{i-1}N(p\left|{{\bar{p}}_{k}}^{i-1},{R_{k}}^{i-1}\right)}$$
4. Following the update of the membership degree parameter, the weight ${\sigma_{K}}^{i}$ is updated. Specifically, the weight of the *k*-th distribution is derived by aggregating the membership degrees of all data points assigned to the *k*-th distribution:
  (15) $${\sigma_{k}}^{i}=\frac{1}{P}\sum_{p}^{P}{\hat{g}}_{k}\left(p\right)$$
5. The center ${{\bar{p}}_{K}}^{i}$ of each distribution is updated by weighting the coordinates p using the integrated normalized amplitude $\bar{I}\left(p\right)$ of the pixel points. The center is then computed by maximizing the expected likelihood based on the membership degree ${\hat{g}}_{k}\left(p\right)$:
  (16) $${{\bar{p}}_{k}}^{i}=\frac{\sum_{p}^{P}{\hat{g}}_{k}\left(p\right)\cdot \bar{I}\left(p\right)\cdot p}{\sum_{p}^{P}{\hat{g}}_{k}\left(p\right)}$$
6. The distribution covariance matrix ${R_{K}}^{i}$ is updated as follows:
  (17) $${R_{k}}^{i}=\frac{\sum_{p}^{P}{\hat{g}}_{k}\left(p\right)\cdot \bar{I}\left(p\right)\cdot \left(p-{{\bar{p}}_{k}}^{i}\right){\left(p-{{\bar{p}}_{k}}^{i}\right)}^{T}}{\sum_{p}^{P}{\hat{g}}_{k}\left(p\right)}$$
7. Steps 3 to 6 are repeated until the weight $\sigma_{K}$, center ${\bar{p}}_{K}$, and distribution covariance matrix $R_{K}$ converge. The convergence condition is defined as:
  (18) $$\left\{\begin{array}{c} {∥{\sigma_{K}}^{i}-{\sigma_{K}}^{i-1}∥}_{1}<\varepsilon_{1}\\ {∥{{\bar{p}}_{K}}^{i}-{{\bar{p}}_{K}}^{i-1}∥}_{1}<\varepsilon_{2}\\ {∥{R_{K}}^{i}-{R_{K}}^{i-1}∥}_{1}<\varepsilon_{3} \end{array}\right.$$

In the formulation, the three convergence thresholds are typically set within 1% to 5% of the parameter magnitudes. For example:

(19) $$\left\{\begin{array}{c} \varepsilon_{1}=0.02\cdot max\left(\sigma_{K}\right)\\ \varepsilon_{2}=0.05\cdot \Delta r\\ \varepsilon_{3}=0.05\cdot {∥{R_{K}}^{0}∥}_{1} \end{array}\right.$$

Once the two-dimensional Gaussian mixture model of the image is obtained, the weights $\sigma_{K}$ are sorted in descending order. The top *M* weights are selected to form the mixture model of the *M* scattering points of the extended target, while the remaining K−M weights are treated as the noise model.

#### 2.2.3. Target Motion Estimation Based on SPS

The scattering distribution of a time-varying extended target may exhibit variations across different processing cycles at distinct time instances, signifying that the positions of the scattering points within the model evolve over time. Effectively estimating the drift values of the target’s scattering points facilitates the acquisition of the latest motion state for robust tracking while simultaneously updating the target model to enhance detection performance.

Assuming that, in the current *t*-th processing cycle, the *M* scattering points of the target have drifted to new positions $a_{m}={\left(x_{m},y_{m}\right)}^{T}$ relative to the previous (t−1)-th processing cycle, the two-dimensional mixture model of the target is modified as follows after accounting for the drift:

(20) $$Gmm\left(p\right)=\sum_{m}^{M}\sigma_{m}N\left(p,|a_{m},R_{m}\right)=\sum_{m}^{M}\sigma_{m}\frac{\exp\left[-\frac{1}{2}{\left(p-a_{m}\right)}^{T}{\left(R_{m}\right)}^{-1}\left(p-a_{m}\right)\right]}{2\pi {\left|R_{m}\right|}^{\frac{1}{2}}}$$

The similarity scale coefficient $\rho^{t}\left(a\right)$ between the drifted model and the image corresponding to the *t*-th processing cycle is defined as follows:

(21) $$\begin{array}{c} \rho^{t}\left(a\right)=\sum_{p}^{P}\sqrt{I^{t}\left(p\right)Gmm\left(p\right)}\\ =\sum_{p}^{P}\sqrt{\sum_{m}^{M}\frac{\sigma_{m}I^{t}\left(p\right)}{2\pi {\left|R_{m}\right|}^{\frac{1}{2}}}\exp\left\{-\frac{1}{2}{\left(p-a_{m}\right)}^{T}{\left(R_{m}\right)}^{-1}\left(p-a_{m}\right)\right\}} \end{array}$$

Subsequently, the positions of the scattering points after drift in the *t*-th cycle are determined by maximizing $\rho^{t}\left(a\right)$:

(22) $$a=\max_{a}\rho^{t}\left(a\right)$$

To maximize $\rho^{t}\left(a\right)$, we need to compute its gradient with respect to $a_{m}$. The gradient of $\rho^{t}\left(a\right)$ with respect to $a_{m}$ is given by:

(23) $$\nabla_{a_{m}}\rho^{t}\left(a\right)=\sum_{p}^{P}\frac{1}{2\sqrt{I^{t}\left(p\right)\cdot Gmm\left(p\right)}}\cdot \nabla_{a_{m}}\mathrm{Gmm}\left(p\right)$$

The gradient of Gmm(p) with respect to $a_{m}$ is:

(24) $$\nabla_{a_{m}}\mathrm{Gmm}\left(p\right)=\sigma_{m}\cdot \frac{\exp\left[-\frac{1}{2}{\left(p-a_{m}\right)}^{T}{\left(R_{m}\right)}^{-1}\left(p-a_{m}\right)\right]}{2\pi {\left|R_{m}\right|}^{\frac{1}{2}}}\cdot {\left(R_{m}\right)}^{-1}\left(p-a_{m}\right)$$

Thus, the gradient of $\rho^{t}\left(a\right)$ becomes:

(25) $$\nabla_{a_{m}}\rho^{t}\left(a\right)=\sum_{p}^{P}\frac{1}{2\sqrt{I^{t}\left(p\right)\cdot Gmm\left(p\right)}}\cdot \sigma_{m}\cdot \frac{\exp\left[-\frac{1}{2}{\left(p-a_{m}\right)}^{T}{\left(R_{m}\right)}^{-1}\left(p-a_{m}\right)\right]}{2\pi {\left|R_{m}\right|}^{\frac{1}{2}}}\cdot {\left(R_{m}\right)}^{-1}\left(p-a_{m}\right)$$

To simplify the optimization, we can use a second-order Taylor expansion of $\rho^{t}\left(a\right)$ around the current estimate of $a_{m}$. The Taylor expansion is given by:

(26) $$\rho^{t}\left(a+\Delta a\right)\approx \rho^{t}\left(a\right)+\nabla_{a_{m}}\rho^{t}{\left(a\right)}^{T}\Delta a+\frac{1}{2}\Delta a^{T}H\left(a\right)\Delta a$$

where H(a) is the Hessian matrix of $\rho^{t}\left(a\right)$ with respect to $a_{m}$. The Hessian can be approximated using numerical methods or computed analytically if feasible.

Using the gradient $\nabla_{a_{m}}\rho^{t}\left(a\right)$, we can perform gradient ascent to iteratively update the target positions $a_{m}$:

(27) $$a_{m}^{\left(k+1\right)}=a_{m}^{\left(k\right)}+\eta \nabla_{a_{m}}\rho^{t}\left(a^{\left(k\right)}\right)$$

where $a_{m}^{\left(k\right)}$ is the estimate of $a_{m}$ at iteration k, η is the learning rate (step size), which can be adjusted dynamically for faster convergence.

The learning rate η is initialized as 0.1·Δr, and adaptively reduced by a factor of 0.5 if $\rho^{t}\left(a\right)$ decreases between iterations. To avoid local minima, we employ a multi-start strategy: the optimization is repeated from three initial positions (previous estimate, perturbed ±Δr), and the solution with the highest $\rho^{t}\left(a\right)$ is selected.

The optimization process stops when the change in $\nabla_{a_{m}}\rho^{t}\left(a\right)$ between iterations falls below a predefined threshold ϵ:

(28) $$\left|\rho^{t}\left(a^{\left(k+1\right)}\right)-\rho^{t}\left(a^{\left(k\right)}\right)\right|<\epsilon$$

The gradient method requires calculations for each pixel in the image. Assuming an image resolution of N×N, the computational complexity for a single gradient calculation is $O(M\times N^{2})$, where *M* represents the number of scattering points. If the number of iterations is *K*, the overall complexity becomes $O(K\times M\times N^{2})$. However, since pixel-level computations are mutually independent, the gradient calculations can be efficiently parallelized on GPU processors. This parallel implementation leads to an exponential reduction in computation time compared to sequential processing.

#### 2.2.4. Likelihood Ratio Detection

After updating the target model, the conditional probability density of each image unit can be obtained under the latest scatter point mixture model and noise model. The conditional probability density of the noise model can be computed as:

(29) $$Gmm\left(p|H_{0}\right)=\frac{\sum_{k=M+1}^{K}\sigma_{k}N(p\left|{\bar{p}}_{k},R_{k}\right)}{\sum_{k=M+1}^{K}\sigma_{k}}$$

The conditional probability density of the scatter point distribution model can be expressed as:

(30) $$Gmm\left(p|H_{1}\right)=\frac{\sum_{k=1}^{M}\sigma_{k}N(p\left|{\bar{p}}_{k},R_{k}\right)}{\sum_{k=1}^{M}\sigma_{k}}$$

By computing the likelihood ratio and performing detection, the positions of the scatter points can be extracted.

## 3. Results and Discussion

### 3.1. Simulation Experiment

To validate the effectiveness of the proposed algorithm, we designed a simulation scenario containing an ET composed of four scattering points.

**Figure 2:**
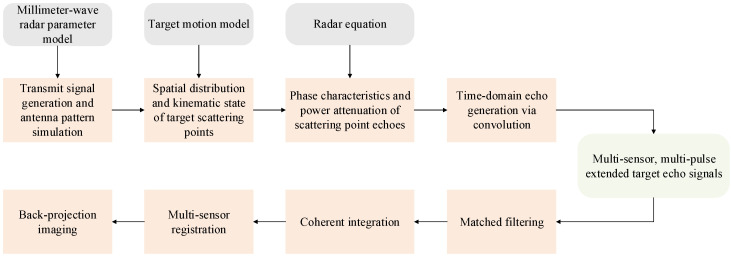
Echo generation and signal preprocessing pipeline for a single CPI.

The simulation experiment encompasses three main components: target echo generation, signal preprocessing, and adaptive tracking/detection. Figure 2 illustrates the computational workflow for echo generation and preprocessing. The pipeline initiates with:

1. Transmitting the signal generation and antenna pattern simulation based on configured radar parameters (carrier frequency, bandwidth, pulse width, PRF, modulation scheme);
2. The determination of scattering point spatial distribution and kinematic states according to the target motion model;
3. The calculation of time delays, phase shifts, and echo power attenuation;
4. The generation of time-domain echoes through the convolution of scattering point distribution with reflection signals;
5. Final output of multi-sensor, multi-pulse echo signals per CPI.

After the echo is generated, the preprocessing stage comprises matched filtering, coherent integration, and multi-sensor registration, culminating in back-projection imaging of the target region.

The imaging region of the simulation scenario is represented by two coordinate axes, x and y, with a distance span of 30 m in both directions. The four scattering points constituting the ET exhibit significant variations in intensity, position, and distribution, with the strong scattering points being more widely distributed compared to the weak ones. The ET as a whole possesses velocity components in both the x and y directions, while each of the four scattering points also exhibits independent motion within the target.

To enhance the realism of the simulation, additive white Gaussian noise (AWGN) with an SNR of 13 dB was superimposed onto the simulated two-dimensional image. The SNR is specifically defined as the peak signal-to-noise ratio. The additive noise is introduced post signal processing to simulate realistic operational conditions.

**Figure 3:**
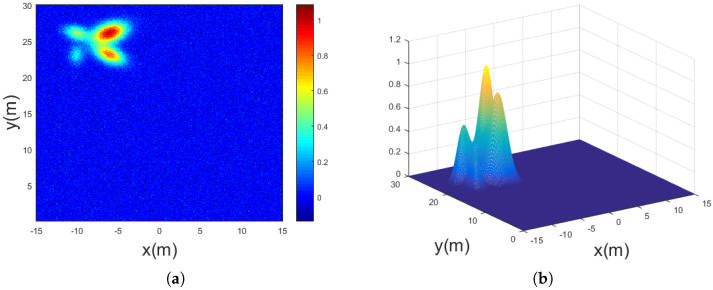
Simulation scenario and processing results. (**a**) The simulated extended target is composed of four scattering points with distinct characteristics. (**b**) The target model initialization results, based on 2D-KDE combined with the Expectation-Maximization algorithm, are presented below.

Figure 3a displays the first frame of the normalized image after the simulation begins. The ET, located in the upper-left region of the image, consists of four scattering points with varying intensities and distributions. Figure 3b illustrates the initial 2D-GMM of the target, obtained through the 2D-KDE algorithm combined with the EM algorithm. The output results and the corresponding simulation parameters are listed in Table 1.

It is evident that the algorithm successfully extracts and accurately models the four scattering points with differing intensities under noisy conditions.

**Figure 4:**
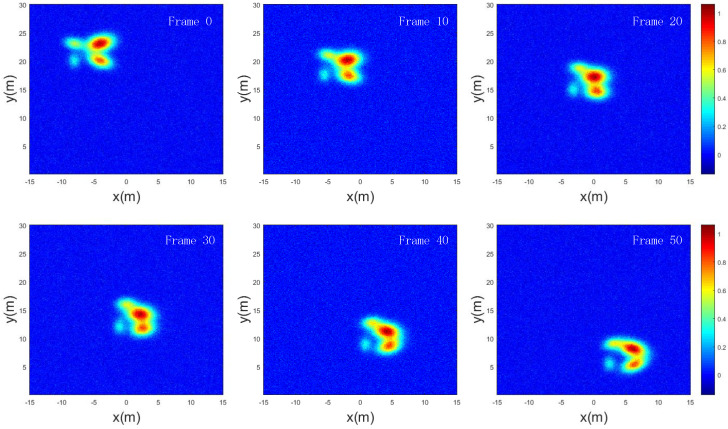
Target motion during the simulation process.

To evaluate the algorithm’s performance in estimating the motion state of the ET and its detection and tracking capabilities, a 50-s simulation scenario was designed based on the aforementioned target parameters. Two-dimensional images were generated and processed at 1-s intervals, resulting in a total of 51 frames. Figure 4 presents the imaging results of the target at the 0th, 10th, 20th, 30th, 40th, and 50th frames. The extended target exhibits a velocity of 0.2 m/s along the x-axis and −0.3 m/s along the y-axis, while each scattering point maintains independent motion within the target. Additionally, the distribution covariance matrix of the scattering points evolves over time.

In addition to the proposed algorithm, we employed KF, PF, PHD, CPHD, VB-BGGIW [25] and CNN-TFD [27] methods for detection and tracking to compare their performance. The position coordinates and velocities of the four scattering points of the extended target, as estimated by these algorithms across multiple frames, were statistically analyzed. The root mean square error (RMSE) of position and velocity estimates was calculated by comparing the results with the ground truth data.

**Figure 5:**
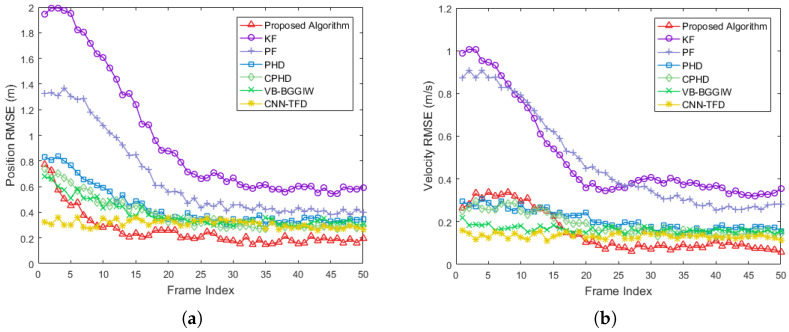
Comparative analysis of multiple algorithms in the simulation experiment. (**a**) Position RMSE. (**b**) Velocity RMSE.

Figure 5 presents a comparative analysis of the processing results from multiple algorithms in the simulation experiment. Specifically, Figure 5a illustrates the RMSE of target position estimation, while Figure 5b shows the RMSE of target velocity estimation. It is evident that the proposed algorithm exhibits faster convergence in both position and velocity estimation compared to the other methods. This is attributed to the elimination of the initial convergence process for filter coefficients in the early frames, which is required by the other algorithms. Furthermore, after convergence, the proposed algorithm achieves lower RMSE values for both position and velocity estimation than the compared methods. Specifically, the proposed algorithm improves position estimation accuracy by 73%, 61%, 50%, 43%, 48% and 42% compared to KF, PF, PHD, CPHD, VB-BGGIW and CNN-TFD respectively. Similarly, it enhances velocity estimation accuracy by 85%, 82%, 67%, 62% 64% and 54% relative to these methods.

**Figure 6:**
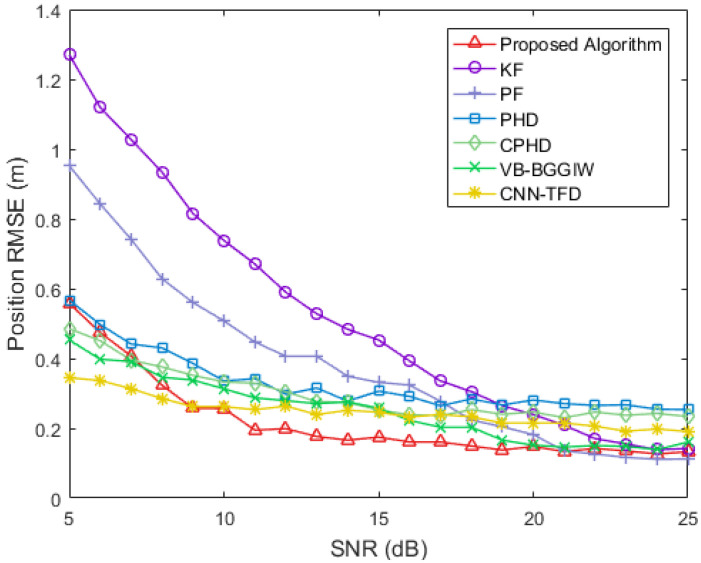
Algorithm stability across varying SNR conditions.

Figure 6 presents a comparative analysis of algorithm stability across varying SNR conditions, illustrating the convergence performance curves of the proposed method against benchmark algorithms within a 5–25 dB SNR range. Experimental results demonstrate that the proposed algorithm achieves superior performance at SNR levels above 10 dB. While its low-SNR performance (below 10 dB) is surpassed by CNN and variational Bayesian methods, it maintains an advantage over both KF and PF approaches.

### 3.2. Field Experiment

**Figure 7:**
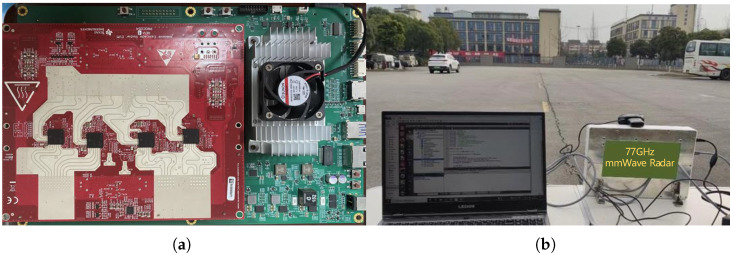
Radar equipment and experimental setup. (**a**) Internal structure of the radar system. (**b**) Experimental scenario for field measurements.

To validate the effectiveness of the proposed algorithm in practical applications, we employed a 77 GHz millimeter-wave radar system, developed using the TI AWR2243 RF front-end, for vehicle target imaging. This radar system transmits the received echo signals to the baseboard processor for digital signal processing. Figure 7 illustrates the radar setup and the experimental scenario. The ground truth target positions were obtained using an IMU-GPS positioning system, with each sensor frame’s timestamp synchronized to determine the target’s true position and motion state for every frame. And the operational parameters of the radar are enumerated in Table 2.

**Figure 8:**
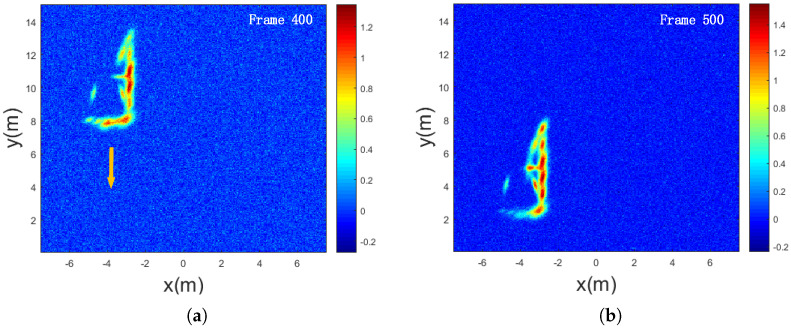
Imaging results with radar positioned at the front-left side of the vehicle. (**a**) Imaging at frame 400. (**b**) Imaging at frame 500.

Due to the occlusion effect caused by the relatively large size of vehicle targets in radar detection, only partial information of the unobstructed regions of the target can be obtained after processing each frame of echo imaging. Figure 8 illustrates the imaging results when the radar is positioned at the front-left side of the vehicle, showing that only the left side, front, and partial upper regions of the vehicle are imaged. In the field experiment, the vehicle traveled at a speed of 10 km/h, and the millimeter-wave radar continuously collected echo data for 10 s, with each coherent processing interval (CPI) lasting 20 ms, resulting in a total of 500 frames. The proposed algorithm, along with four other algorithms, was applied to process the echo data, and the results were analyzed.

**Figure 9:**
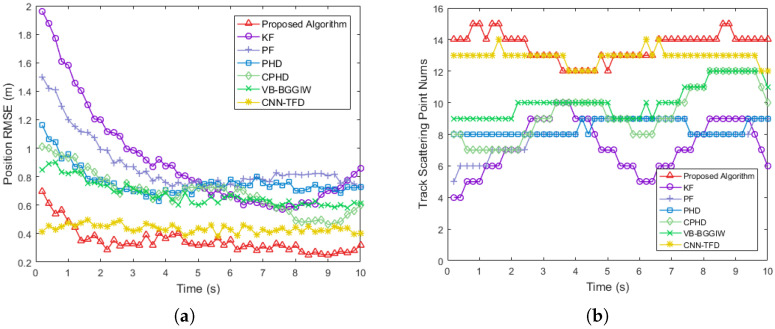
Comparative analysis of multiple algorithms in the field experiment. (**a**) Position RMSE. (**b**) Number of tracked scattering points.

Figure 9 presents the evaluation results of the field experiment. Specifically, Figure 9a illustrates the position root mean square error (RMSE) of the detected vehicle using five different algorithms, while Figure 9b provides a statistical comparison of the number of scattering points processed in real-time using these algorithms over the 10-s duration. The radar was configured with a signal bandwidth of 1 GHz, yielding a range resolution of 0.15 m. As shown, the proposed algorithm achieves a position RMSE between 0.2 m and 0.25 m after convergence, which is close to the radar’s range resolution. In contrast, the position RMSE of the other four algorithms significantly exceeds the range resolution, resulting in suboptimal tracking performance. Figure 9b elucidates the underlying reasons for this phenomenon: the proposed algorithm not only demonstrates excellent processing capabilities for strong scattering points but also effectively extracts weak scattering points. From the first frame, it maintains a high number of processed scattering points, thereby significantly improving the utilization of echo information and enabling accurate estimation of the vehicle’s position. Conversely, the other four algorithms tend to lose partial information of weak scattering points during processing, leading to considerable errors in target estimation.

The proposed signal preprocessing pipeline and detection algorithm were implemented on GPU architecture to achieve real-time processing capabilities. Specifically, our millimeter-wave radar system incorporates an NVIDIA TX2 GPU processor (NVIDIA Corporation, Santa Clara, CA, USA), enabling a processing cycle of 20 ms (equivalent to 50 Hz). Table 3 presents a comparative analysis of computational efficiency, detailing the processing times required by the proposed method and several benchmark algorithms when operating on experimental datasets.

## 4. Conclusions

In this study, we introduced a novel algorithm for adaptive detection and tracking of extended targets utilizing a millimeter-wave imaging radar, which enables precise and robust detection and tracking of non-cooperative extended targets. By exploiting the statistical properties of ETs in radar imaging, a 2D-GMM was constructed to accurately characterize the scattering distribution of most targets in high-resolution radar sensing scenarios. Under low SNR conditions, the proposed approach, which integrates 2D-KDE with expectation maximization, effectively captures the distribution features of weak scattering points. The accuracy of the target model parameter estimation is confirmed through extensive simulation experiments. In comparison to conventional filtering techniques and RFS-based methods, the proposed scattering point drift-based target motion estimation algorithm demonstrates superior convergence speed and reduced tracking error, as evidenced by real-world experimental results. Furthermore, the tracking precision of the proposed algorithm for complex dynamic ETs approaches the radar’s range resolution. The combined results from both simulation and real-world experiments validate the effectiveness and practical applicability of our proposed method.

This study has several inherent limitations that warrant discussion. The primary focus of the current work is to establish an adaptive detection and tracking framework for extended targets based on the Scattering Point Shift principle. The main objective is to validate the fundamental feasibility of the proposed framework for extended target scenarios. Consequently, this investigation does not account for environments with high clutter interference. Future research will address the challenges posed by multi-target scenarios, intense clutter conditions, and even multipath interference to enhance the algorithm’s applicability in real-world operational environments.

## Figures and Tables

**Table 1:** Output results of 2D-KDE algorithm.

Parameter	True Values	Estimated Values
Scattering point 1		
Peak value	1.0	1.0
Central position	[−6, 26]	[−6, 26]
Covariance matrix	[2 0.5; 0.5 1]	[1.98 0.53; 0.51 0.99]
Scattering point 2		
Peak value	0.5	0.51
Central position	[−10, 26]	[−9.9, 25.9]
Covariance matrix	[1 −0.2; −0.2 0.5]	[0.96 −0.2; −0.19 0.5]
Scattering point 3		
Peak value	0.8	0.81
Central position	[−6, 23]	[−6, 23.1]
Covariance matrix	[1.5 −0.5; −0.5 0.8]	[1.51 −0.49; −0.5 0.78]
Scattering point 4		
Peak value	0.3	0.29
Central position	[−10, 23]	[−9.8, 23]
Covariance matrix	[0.4 0; 0 0.8]	[0.38 0.03; 0.01 0.83]

**Table 2:** mm-Wave radar operational parameters.

Symbol	Parameter	Value
$f_{c}$	Carrier frequency	76–80 GHz
*B*	Bandwidth of the signal	1 GHz
$f_{s}$	Sampling rate	1.5 GHz
CPI	Coherent processing interval	20 ms

**Table 3:** Computational times comparison.

Method	Computational Times (ms)
SPS	15.7
KF	5.1
PF	204.4
PHD	241.6
CPHD	288.7
VB-BGGIW	183.5
CNN-TFD	325.2

## Data Availability

The original contributions presented in the study are included in the article material; further inquiries can be directed to the corresponding author.

## Abbreviations

The following abbreviations are used in this manuscript:

ETs	Extended targets
SPS	Scattering Point Shift
2D-KDE	Two-dimensional kernel density estimation
mmWave	Millimeter-wave
LFMCW	Linear Frequency Modulated Continuous Wave
KF	Kalman Filter
PF	Particle filter
RFS	Random Finite Set
ETT	Extended Target Tracking
PHD	Probability Hypothesis Density
CPHD	Cardinalized Probability Hypothesis Density
2D-GMM	Two-dimensional Gaussian mixture model
CPI	Coherent processing interval
SNR	Signal-to-noise ratio
EM	Expectation-Maximization
AWGN	Additive white Gaussian noise
RMSE	Root mean square error

## References

[1] Gao X., Roy S., Xing G. (2021). MIMO-SAR: A Hierarchical High-Resolution Imaging Algorithm for mmWave FMCW Radar in Autonomous Driving. IEEE Trans. Veh. Technol..

[2] Kosuge A., Suehiro S., Hamada M., Kuroda T. (2022). mmWave-YOLO: A mmWave Imaging Radar-Based Real-Time Multiclass Object Recognition System for ADAS Applications. IEEE Trans. Instrum. Meas..

[3] Zhang Y., Liu Q., Hong R. (2016). A Novel Monopulse Angle Estimation Method for Wideband LFM Radars. Sensors.

[4] Richards M.A. (2014). Fundamentals of Radar Signal Processing.

[5] Jiao H., Yan J., Pu W., Chen Y., Liu H., Greco M.S. (2025). Wideband Sensor Resource Allocation for Extended Target Tracking and Classification. IEEE Trans. Signal Process..

[6] Liu M., Zhao K., Zhang Y., Gao Y., Zhang T. Feature Aided Extended Target Tracking For High Resolution Radar. Proceedings of the 2021 33rd Chinese Control and Decision Conference (CCDC).

[7] Wang S., Men C., Li R., Yeo T.-S. (2024). A Maneuvering Extended Target Tracking IMM Algorithm Based on Second-Order EKF. IEEE Trans. Instrum. Meas..

[8] Sang H., Zheng R., Cheng H., Meng X., Li L., Qi J. A Review of Point Target and Extended Target Tracking Algorithms. Proceedings of the 2024 3rd International Conference on Image Processing and Media Computing (ICIPMC).

[9] Brooker G. (2013). Introduction to Sensors for Ranging and Imaging.

[10] Liu Q., Cheng Y., Cao K., Liu K. (2023). Radar 3-D Forward-Looking Imaging for Extended Targets Based on Attribute Scattering Model. IEEE Geosci. Remote Sens. Lett..

[11] Yoon Y.S., Amin M.G. High resolution through-the-wall radar imaging using extended target model. Proceedings of the 2008 IEEE Radar Conference.

[12] Liu B., Tharmarasa R., Jassemi R., Brown D. (2023). RFS-Based Multiple Extended Target Tracking with Resolved Multipath Detections in Clutter. IEEE Trans. Intell. Transp. Syst..

[13] Zhong Z., Meng H., Wang X. Extended target tracking using an IMM based Rao-Blackwellised unscented Kalman filter. Proceedings of the 2008 9th International Conference on Signal Processing.

[14] Xu J. (2017). Extended Target Tracking Using a Gaussian Mixture Model and Kalman Filter. IEEE Trans. Signal Process..

[15] Liu H. (2016). Extended Target Tracking Using Gaussian Mixture Model and Particle Filter. IEEE Trans. Aerosp. Electron. Syst..

[16] Wu W., Liu D., Sun J. (2024). Multihypothesis Multimodel Elliptic Radon Transform for Low-Observability Maneuvering Range-Ambiguity Target Detection. IEEE Trans. Aerosp. Electron. Syst..

[17] Yang S., Baum M. Metrics for performance evaluation of elliptic extended object tracking methods. Proceedings of the 2016 IEEE International Conference on Multisensor Fusion and Integration for Intelligent Systems (MFI).

[18] Shen X., Song Z., Fan H. PHD filter for single extended target tracking. Proceedings of the 2016 CIE International Conference on Radar (RADAR).

[19] Beard M., Reuter S. (2016). Multiple Extended Target Tracking with Labeled Random Finite Sets. IEEE Trans. Signal Process..

[20] Granstrom K., Lundquist C., Orguner O. (2012). Extended Target Tracking using a Gaussian-Mixture PHD Filter. IEEE Trans. Aerosp. Electron. Syst..

[21] Chen Y., Liu W. Multiple extended target tracking based on GLMB filter and gibbs sampler. Proceedings of the 2017 International Conference on Control, Automation and Information Sciences (ICCAIS).

[22] Eryildirim A., Guldogan M.B. (2016). A Bernoulli Filter for Extended Target Tracking Using Random Matrices in a UWB Sensor Network. IEEE Sens. J..

[23] Yuan Y., Ma S. Joint Multi-Target Tracking and Identification for Distributed Radars Using Bayesian Binary Test. Proceedings of the 2024 IEEE Radar Conference.

[24] Beard M. (2015). Bayesian Multi-Target Tracking with Merged Measurements Using Labelled Random Finite Sets. IEEE Trans. Signal Process..

[25] Yang X., Jiao Q. Variational Approximation for Extended Target Tracking in Clutter with Random Matrix. Proceedings of the 2021 International Conference on Control, Automation and Information Sciences (ICCAIS).

[26] Zhao L. (2020). Deep Learning for Radar Target Detection and Tracking: A Survey. IEEE Trans. Signal Process..

[27] Ding M., Ding Y., Peng Y. (2023). CNN-Based Time–Frequency Image Enhancement Algorithm for Target Tracking Using Doppler Through-Wall Radar. IEEE Geosci. Remote Sens. Lett..

[28] Zhu Y. UAV Trajectory Tracking via RNN-Enhanced IMM-KF with ADS-B Data. Proceedings of the 2024 IEEE Wireless Communications and Networking Conference (WCNC).

[29] Zhang Y. (2018). Robust Extended Target Tracking in Cluttered Environments. IEEE Trans. Aerosp. Electron. Syst..

[30] Huang X. (2019). Multi-target Tracking of Extended Targets Using a Hybrid Filter. IEEE Trans. Radar Syst..

[31] Seng C.H., Bouzerdoum A., Amin M.G., Ahmad F. A Gaussian-Rayleigh mixture modeling approach for through-the-wall radar image segmentation. Proceedings of the 2012 IEEE International Conference on Acoustics, Speech and Signal Processing (ICASSP).

[32] Kilaru V., Amin M.G., Ahmad F., Sévigny P., DiFilippo D. Gaussian mixture model based features for stationary human identification in urban radar imagery. Proceedings of the 2014 IEEE Radar Conference.

